# Chemotherapy Plus Atezolizumab Pre- and Post-Resection in Localized Esophageal or Gastroesophageal Junction Adenocarcinomas: A Phase I/II Single-Arm Study

**DOI:** 10.3390/cancers16071378

**Published:** 2024-03-31

**Authors:** Matheus Sewastjanow-Silva, Lianchun Xiao, Graciela N. Gonzalez, Xuemei Wang, Wayne Hofstetter, Stephen Swisher, Reza Mehran, Boris Sepesi, Manoop S. Bhutani, Brian Weston, Emmanuel Coronel, Rebecca E. Waters, Jane E. Rogers, Jackie Smith, Larry Lyons, Norelle Reilly, James C. Yao, Jaffer A. Ajani, Mariela Blum Murphy

**Affiliations:** 1 Department of Gastrointestinal Medical Oncology, The University of Texas MD Anderson Cancer Center, Houston, TX 77030, USA; msewastjanow@mdanderson.org (M.S.-S.); jsmith19@mdanderson.org (J.S.); jyao@mdanderson.org (J.C.Y.); jajani@mdanderson.org (J.A.A.); 2 Department of Biostatistics, The University of Texas MD Anderson Cancer Center, Houston, TX 77030, USA; lxiao@mdanderson.org (L.X.); gnoguera@mdanderson.org (G.N.G.); xuewang@mdanderson.org (X.W.); 3 Department of Thoracic and Cardiovascular Surgery, The University of Texas MD Anderson Cancer Center, Houston, TX 77030, USA; whofstetter@mdanderson.org (W.H.); sswisher@mdanderson.org (S.S.); rjmehran@mdanderson.org (R.M.); bsepesi@mdanderson.org (B.S.); 4 Department of Gastroenterology, Hepatology and Nutrition, The University of Texas MD Anderson Cancer Center, Houston, TX 77030, USA; manoop.bhutani@mdanderson.org (M.S.B.); bweston@mdanderson.org (B.W.); ecoronel@mdanderson.org (E.C.); 5 Department of Pathology, The University of Texas MD Anderson Cancer Center, Houston, TX 77030, USA; rwaters@mdanderson.org; 6 Department of Pharmacy Clinical Programs, The University of Texas MD Anderson Cancer Center, Houston, TX 77030, USA; jerogers@mdanderson.org; 7 Genentech Inc., South San Francisco, CA 94080, USAreilly.norelle@gene.com (N.R.)

**Keywords:** adenocarcinoma, atezolizumab, esophagus, gastroesophageal junction, immunotherapy, NCT03784326, trial

## Abstract

**Simple Summary:**

What is the efficacy and safety of atezolizumab with oxaliplatin and 5-fluorouracil followed by atezolizumab for localized esophageal or gastroesophageal junction adenocarcinomas before surgical resection? In this non-randomized controlled clinical trial, the observed pathological complete response (downstaging to ypT0N0M0) after surgery was 11.1%. The median overall survival was 38.6 months, and the median disease-free survival was 28.8 months. Two patients had grade 4 treatment-related adverse events. These findings suggest that atezolizumab with oxaliplatin and 5-fluorouracil for the treatment of localized, resectable adenocarcinoma of the esophagus or gastroesophageal junction showed moderate efficacy with an acceptable safety profile and warrants further investigation.

**Abstract:**

Efforts to improve the prognosis for patients with locally advanced esophageal or gastroesophageal junction (GEJ) adenocarcinoma have focused on neoadjuvant approaches to increase the pathological complete response (pathCR) rate, improve surgical resection, and prolong event-free and overall survival (OS). Building on the recent evidence that PD-1 inhibition plus chemotherapy improves the OS of patients with metastatic GEJ adenocarcinoma, we evaluated whether the application of this strategy in the neoadjuvant setting would improve the pathological response. This single-center phase I/II trial evaluated the safety, toxicity, and efficacy of neoadjuvant atezolizumab with oxaliplatin and 5-fluorouracil (modified FOLFOX) followed by esophagectomy followed by atezolizumab. The primary objective goal was to achieve 20% pathCR. From the twenty enrolled patients, eighteen underwent resection and two (10%, 95% CI: 1.24–31.7%) achieved pathCR. After a median follow-up duration of 40.7 months, 11 patients had disease recurrence and 10 had died. The median disease-free and OS were 28.8 (95% CI: 14.7, NA) and 38.6 months (95% CI: 30.5, NA), respectively. No treatment-related adverse events led to death. Although modified FOLFOX plus atezolizumab did not achieve the expected pathCR, an acceptable safety profile was observed. Our results support the continued development of a more refined strategy (neoadjuvant chemotherapy plus perioperative immunotherapy/targeted agents) with molecular/immune profiling in parallel.

## 1. Introduction

Esophageal cancer (EC) is a disease with a poor overall survival rate and very limited options for treatment. The aggressive biology of this disease, typically already in an advanced stage at the time of diagnosis, and resistance to conventional treatments are factors that contribute to the dismal outcomes. A limited proportion of patients are found to have resectable disease at diagnosis; even after local treatment of the primary tumor, survival at 5 years after diagnosis ranges from 48.8% in localized disease to 5.6% if metastatic [1,2]. Therefore, the management of EC has focused on neoadjuvant approaches that aim to increase the pathological complete response (pathCR) rate, improve resectability, and ultimately, increase overall survival (OS).

Neoadjuvant therapy (chemotherapy or chemoradiation) followed by surgery is currently considered the standard of care in resectable locally advanced EC [3,4]. While chemotherapy, immunotherapy, and/or targeted therapy are typical approaches in the context of metastatic disease, several of these strategies are currently being studied in the localized setting. Most recently, CheckMate 577 established the benefit of adding a Programmed death-1 (PD-1) inhibitor in the adjuvant setting after neoadjuvant chemoradiation and surgery in patients with residual pathological disease (i.e., absence of pathological complete response) [5]. The results from this study demonstrated improved disease-free survival (DFS) with adjuvant PD-1 inhibition compared with the placebo, setting a new standard of care. At the time of study conception, there was an increased interest in the idea that the combination of chemotherapy and immunotherapy in the neoadjuvant setting for patients with localized esophageal and gastroesophageal junction (GEJ) adenocarcinomas had the potential to increase disease control and to prolong survival.

Atezolizumab is a humanized immunoglobulin (Ig) G1 monoclonal antibody that targets PD-L1 (Programmed death-ligand 1) and blocks its interaction with its receptors, PD-1 and B7-1 (also known as CD80), both of which are inhibitory receptors expressed on T cells. This immune checkpoint inhibitor (ICI) agent has been shown to increase the magnitude and quality of tumor-specific T-cell responses, resulting in better anti-tumor efficacy by helping to prevent the inhibitory signals that can suppress immune activity [6,7]. Atezolizumab is currently FDA-approved for patients with unresectable or metastatic hepatocellular carcinoma, previously treated locally advanced or metastatic non-small-cell lung cancer (NSCLC), extensive-stage small-cell lung cancer (SCLC), alveolar soft part sarcoma, and unresectable or metastatic melanoma. Furthermore, it is also recommended for the treatment of patients with recurrent or metastatic small-cell neuroendocrine carcinoma of the cervix, advanced triple-negative breast cancer with PD-L1 expression in ≥1% tumor-infiltrating immune cells and for metastatic urothelial carcinoma patients who are ineligible for any platinum-containing chemotherapy irrespective of the PD-L1 expression in their tumors or who are ineligible for cisplatin chemotherapy [8,9,10,11].

Our study (NCT03784326) combined systemic chemotherapy with atezolizumab in the neoadjuvant treatment of patients with localized esophageal/GEJ adenocarcinomas. Patients underwent esophagectomy after completion of neoadjuvant treatment and subsequently received adjuvant atezolizumab. Here, we present the safety and efficacy results of this trial.

## 2. Patients and Methods

We conducted a prospective, single-institution, phase I/II trial at the University of Texas MD Anderson Cancer Center (MD Anderson) in Houston, TX (National Clinical Trial identifier: NCT03784326). The MD Anderson Institutional Review Board approved this study. Written consent was obtained from all patients before enrollment. This study was conducted in accordance with the Declaration of Helsinki (1964).

The following criteria were required for study entry: (1) age ≥ 18 years at the time the informed consent document was signed; (2) histologically or cytologically confirmed esophageal or gastroesophageal Siewert type I or II adenocarcinoma; (3) no prior therapy, including chemotherapy or radiation therapy; (4) resectable, T1N1, and T2–3 stage with any N+ at the time of screening; (5) ECOG performance status of 0–1; (6) adequate baseline bone marrow function (neutrophils > 1.5 × 10^9^/L and platelets > 100 × 10^9^/L), hepatic function [bilirubin < 1.5 times upper limit of the normal range (ULN), AST and/or ALT < 2.5 times ULN], and renal function (serum creatinine, 1.5 times ULN); (7) medical fitness for surgery based on surgeon assessment; and (8) no evidence of HIV, hepatitis B, or hepatitis C infection.

Major exclusion criteria: (1) T1aN0, T4b, or M1 stage; (2) squamous cell carcinoma histology; (3) significant comorbid conditions (such as uncontrolled hypertension or diabetes, heart failure, or ongoing mental health issue that prevents compliance and consistent participation in the clinical trial); (4) inability to comprehend or comply with the requirements of this study; (5) primary tumor hemorrhage demanding radiation therapy control; (6) neuropathy ≥ grade 1; (7) symptomatic or uncontrolled hypercalcemia (calcium > 12 mg/dL, Ca^2+^ > 1.5 mmol/L, or corrected serum calcium > upper limit of normal); (8) patients with current or past immunological diseases; and (9) history of idiopathic pulmonary fibrosis, organizing pneumonia (e.g., bronchiolitis obliterans), or drug-induced pneumonitis.

**Figure 1:**
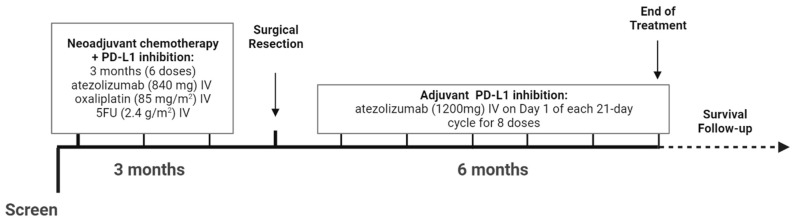
Summary of treatment regimen. Created at Biorender.com on 20 October 2023.

Prior to surgical resection, patients received atezolizumab (840 mg) intravenously (IV) in combination with oxaliplatin (85 mg/m^2^) IV and 5-fluorouracil (5FU) (2.4 g/m^2^) IV on Days 1 and 15 of each 28-day cycle for a total of 6 doses (Figure 1). After surgery, patients received adjuvant atezolizumab (1200 mg) IV on Day 1 of each 21-day cycle for 8 doses, as seen in Figure 1. Atezolizumab was given until treatment completion or discontinued if any unacceptable toxicity or loss of clinical benefit was observed after an integrated assessment of radiographic and biochemical data, local biopsy results (if these were available), and clinical status.

The primary objective of this study was to estimate pathCR (ypT0N0M0) in the surgical specimen following neoadjuvant atezolizumab in combination with modified FOLFOX therapy; the goal was to achieve at least 20% pathCR. The secondary and exploratory objectives were to evaluate the safety and toxicity profiles of this neoadjuvant regimen, DFS and OS rates, overall safety and tolerability of adjuvant atezolizumab, efficacy of this regimen by assessing tumor regression grade scoring in the surgical sample, and changes in tumor stroma profile before and after treatment.

Pre-treatment and post-resection cancer staging was performed according to the eighth edition of the American Joint Committee on Cancer staging manual. Toxicity was graded according to the National Cancer Institute Common Toxicity Criteria Version 5.0.

Descriptive statistics and frequency tables were used to characterize the study population and summarize the adverse event (AE) and outcome data. The Kaplan–Meier method was used to estimate the probability of the time-to-event outcomes, i.e., OS and DFS. OS was calculated as the time from the date of diagnosis to the date of death; patients still alive at the end of follow-up were censored on the date of last follow-up. DFS was calculated as time from the date of surgery to the date of recurrence, death, or last known follow-up, whichever occurred first. Statistical analyses were performed using Stata/SE version 16.1 statistical software (Stata Corp. LP, College Station, TX, USA).

## 3. Results

Between April 2019 and November 2020, 20 patients were enrolled. The median age was 60.9 years (range: 39.9–79.1), and eight patients were aged 65 years or older. Most patients were White (95%), non-Hispanic (80%), and male (85%), with a T3 primary (95%) and a performance status of 1 (60%) (Table 1). Overall, 50% of tumors were poorly differentiated and 60% were localized in the distal part of the esophagus (Siewert I); 40% had a signet-ring-cell component.

Eighteen patients had surgery, one had early progression, and one declined surgery due to neoadjuvant treatment toxicity. Eight patients received adjuvant atezolizumab; the reasons for not completing adjuvant therapy were poor response observed during surgical pathological examination (*n* = 7), toxicity (*n* = 2), and patient decision to withdraw from this study (*n* = 1).

Out of the twenty patients enrolled, two patients [10%, 95% confidence interval (CI): 1.24–31.7%] achieved pathCR (ypT0N0M0; Table 2). However, eighteen patients successfully underwent surgical resection, four patients (22%) achieved major pathological response (<1% viable tumor in surgical samples), and two achieved pathCR (11%). While 15 (83.3%) patients had R0 resection (no tumor within 1 mm of the resection margins), ≥30% residual tumor and lymphovascular invasion were seen in 14 and 11 patients, respectively. A median of 33 lymph nodes were resected; eight (44.4%) patients had ≥1 positive lymph nodes in the resection specimen. Out of the 18 patients who had complete staging results before and after surgery, 12 had a decrease in their staging (downstaged). Nodal downstaging was observed in seven patients; additionally, four patients were T0 pre-treatment as well as after surgical resection. Out of the eight patients who received adjuvant atezolizumab, two had local recurrence, and one progressed with metastatic disease to the liver.

### 3.1. Survival

**Figure 2:**
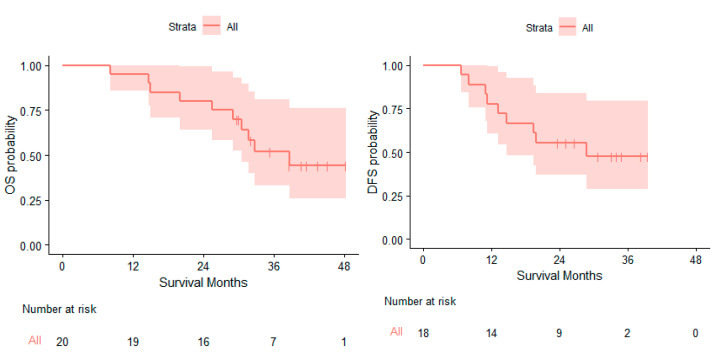
Kaplan–Meier curves for overall survival and disease-free survival. Median overall survival: 38.6 months (95% CI: 30.5, NA); median disease-free survival: 28.8 months (95% CI: 14.7, NA).

At a median follow-up time of 40.7 months (95% CI: 35.3, NA), 10 of the 20 patients had died. The median OS (mOS) was 38.6 months (95% CI: 30.5, NA). Ten of the eighteen patients who underwent surgery had disease recurrence or died. The median DFS (mDFS) was 28.8 months (95% CI: 14.7, NA) (Figure 2).

### 3.2. Toxicity

Adverse events (AEs) of any grade occurred in 18 of 20 patients (90%), and none of these were grade 5. Grade 3 or 4 AEs occurred in nine patients (45%); the most common grade 3 and 4 AEs were fatigue [four patients (20%)] and pancreatitis [two patients (10%)]. Two grade 4 AEs were observed (Table 3). A patient with a history of supraventricular tachycardia had a grade 4 hypertensive crisis in the third cycle, deemed as definitely related to atezolizumab, and was taken out of this study. One case of grade 4 pancreatitis related to atezolizumab was observed in another patient; it resolved in 7 days after treatment with steroids. Seven patients presented grade 3 AEs related to atezolizumab, fatigue being the most frequent (Table 4). Table 4 shows the number of patients who had grade 3 or 4 AEs related to atezolizumab.

## 4. Discussion

Attempts to improve the currently dismal prognosis in EC and GEJ cancers are currently focused on neoadjuvant approaches to reduce tumor burden and increase resectability. The current standard-of-care treatment for localized EC or GEJ (Siewert I or II) adenocarcinoma includes neoadjuvant chemotherapy or neoadjuvant chemoradiation (nCRT) prior to surgery. While different neoadjuvant chemotherapy regimens have been investigated (CROSS [12], FLOT4 [13], MAGIC [14], and OE05 [15] trials), with different toxicity profiles observed, the standard backbone includes platinum and fluoropyrimidine. Given recent evidence demonstrating that PD-L1 inhibition may improve anti-tumor activity in the adjuvant setting, we conducted a single-center phase I/II trial combining chemotherapy and immunotherapy for the neoadjuvant treatment of localized esophageal and GEJ (Siewert I or II) cancer.

At the time of study design, the standard systemic chemotherapy regimen for esophageal cancer included platinum and fluoropyrimidine. Still, fluoropyrimidine and oxaliplatin continues to be a preferred regimen in the National Comprehensive Cancer Network (NCCN) guidelines [3].

In the CROSS trial, the addition of neoadjuvant radiation was associated with improved rates of nodal downstaging to ypN0 (60.1% vs. 44.5%, *p* = 0.004), R0 resection/negative margins (95% vs. 82%, *p* < 0.001), major pathologic response rate (42% vs. 12%, *p* < 0.001), and pathologic complete response (17% vs. 5%, *p* = 0.001). However, the final primary outcome analysis from the Neo-Aegis study [4] showed no evidence of an OS advantage in perioperative chemotherapy versus nCRT [3-year OS: 55% vs. 57%, hazard ratio (HR) = 1.03, 95% CI: 0.77–1.38] and a respective mOS of 48 months (95% CI: 33.6–64.8) and 49.2 months (95% CI: 34.8–74.4). Additionally, the PERFECT trial [16], which investigated the role of nCRT combined with atezolizumab for resectable esophageal adenocarcinomas, did not show a statistically significant improvement in response (R0 resection: PERFECT 82.5% vs. nCRT 85.8%, *p* = 0.61; pathCR: PERFECT 25% vs. nCRT 20.1%, *p* = 0.51; or Mandard tumor regression grade (TRG) 1–2: PERFECT 37.5% vs. nCRT 43.3%, *p* = 0.52) or survival (mOS: PERFECT 29.7 months vs. nCRT 34.3 months, HR = 0.78, 95% CI, 0.42–1.45, *p* = 0.43) despite its noteworthy superior, but not statistically significant, observed pathCR rate.

Since the completion of this study, a similar clinical trial has been published: the PANDA trial [17] has shown the potential for the use of neoadjuvant atezolizumab plus chemotherapy in nonmetastatic, resectable gastric and gastroesophageal junction adenocarcinomas by presenting a 45% pathCR rate (defined as 0% residual viable tumor, Mandard TRG1, regardless of lymph node status) and that 14 out of 20 patients were alive at the median follow-up time of 47 months. There are no available data on how many patients were ypT0N0M0. Additional immunological analyses after the first dose of atezolizumab showed that PD-1^+^ CD8^+^ T-cell infiltration was significantly higher in responders and that these had lower levels of circulating tumor DNA. Immune activation in the tumor microenvironment was thus observed.

Other than the tumor location differences, important distinctions between our trial and PANDA were their strategy of including only atezolizumab in the preoperative first cycle, without any chemotherapeutic agent, followed by four cycles of atezolizumab combined with docetaxel, oxaliplatin, and capecitabine. Moreover, we observed in our study a higher prevalence of more aggressive [18] tumor histology (50% poorly differentiated/40% SRCs vs. 19% diffuse/mixed at PANDA) and a lower prevalence of deficient mismatch repair (dMMR/MSI-H) tumors (0% dMMR vs. 14% dMMR at PANDA). MSI-H tumors are known to have a better response [19] when treated with immune checkpoint inhibitors; this might explain part of the achievement of a high pathCR rate in the PANDA trial. That cohort was also favored by the 90% prevalence of patients with a positive PD-L1 result (CPS ≥ 1), thus responding very well to the PD-L1 inhibitor [20], while 35% of our cohort (*n* = 7/20 patients) had a positive PD-L1 result. A larger randomized controlled clinical trial is necessary to not only reduce biases but to warrant validation of these observed results.

Similarly to PANDA, the DANTE trial [21,22] investigated the use of perioperative atezolizumab with chemotherapy in resectable gastric or gastroesophageal junction adenocarcinomas and also had a larger percentage of PD-L1-positive (58%) and MSI-H (8%) patients, showed downstaging benefits (ypT0, 23% vs. 15%, *p* = 0.044; ypT0-T2, 61% vs. 48%, *p* = 0.015; ypN0, 68% vs. 54%, *p* = 0.012). It is interesting to note though that the atezolizumab arm had a considerably lower prevalence of diffuse type (16% vs. 30%). PathCR plus pathologic complete regression (TRG 1a) percentages were higher in the atezolizumab group (24% vs. 15%; *p* = 0.032), with this difference being more pronounced in the PD-L1 CPS ≥ 10 (33% vs. 12%) and MSI-H (63% vs. 27%) subpopulations. In summary, these show the advantages of this treatment strategy in a population with a higher prevalence of both biomarkers, yet survival analyses are awaited. Their toxicity data showed an increased number of grade 3–4 infections in the atezolizumab arm.

Immune checkpoint inhibitors have been found to improve OS when added to front-line chemotherapy in patients with advanced esophageal, GEJ, and gastric cancer [20,23,24,25]. However, the current results from other trials incorporating ICIs in the localized setting, namely Attraction-5 [26] (post-operative approach but relevant to occult disease) and Keynote-585 [27], are mostly not superior. Attraction-5 evaluated the role of nivolumab plus chemotherapy in 755 Asian patients with GEJ or gastric cancer whose post-operative staging was stage III, though the primary efficacy endpoint of centrally assessed relapse-free survival (RFS) was not met (HR: 0.90; 95.72% CI: 0.69–1.18; *p* = 0.4363), with 3-year RFS rates of 68.4% (95% CI: 63.0–73.2) in the nivolumab plus chemotherapy arm and 65.3% (95% CI: 59.9–70.2) in the chemotherapy alone arm. Similarly, Keynote-585 evaluated the role of another ICI, pembrolizumab, with chemotherapy for localized GEJ or gastric adenocarcinomas, and the preliminary results demonstrated a statistically significant improvement in pathCR when chemotherapy was combined with pembrolizumab; however, no statistical significance was observed for event-free survival (EFS) and OS [28]. Taken together with the results from this current trial, which presents novelty by including esophageal adenocarcinomas, the combination of current ICIs + chemotherapy is tolerable but does not seem to be enough to provide a statistically significant improvement in path CR or survival for patients with esophagogastric adenocarcinomas.

Newer ICI agents such as durvalumab and toripalimab have shown encouraging results in gastric and gastroesophageal junction cancers [20,29,30,31]. Preliminary results from the MATTERHORN (phase 3) trial showed improved pathCR (19% vs. 7%, odds ratio [OR] 3.08; *p* < 0.00001) when durvalumab was combined with neoadjuvant FLOT versus FLOT alone for resectable gastric and GEJ cancers; EFS and OS results are still forthcoming. It is important to note that the control arm of the MATTERHORN trial, where 474 patients received neoadjuvant FLOT, achieved only 7% pathCR.

Similarly, early analysis of a phase II study of toripalimab plus chemotherapy in the context of perioperative treatment of resectable gastric or GEJ adenocarcinomas also showed improved pathCR [24.1% (13/54, 95% CI: 13.5–37.6%) vs. 9.3% (5/54, 95% CI: 3.1–20.3%); *p* = 0.039] and downstaging [ypT0–2: 46.3% vs. 22.2% (*p* = 0.002); yp stage 0–1: 38.9% vs. 16.7% (*p* = 0.024)] [30]. Given the known potential of ICIs, we are exploring the addition of tiragolumab, a T-cell immunoreceptor with Ig and ITIM domains (TIGIT) inhibitor, to atezolizumab and standard chemotherapy in another arm of this current clinical trial to further understand the effects of the concurrent use of ICIs with different mechanisms of action/target domains in esophageal and gastroesophageal junction adenocarcinomas, as this combination had shown positive results in lung cancer [32].

### Limitations

Although our observed pathCR rate (10%, 95% CI: 1–28%), our primary endpoint, was lower than hypothesized, the relatively low number of enrolled patients reduced the confidence interval precision and statistical power. Biomarker tests for HER2, PD-L1, and microsatellite status (MS) were usually requested in the setting of metastatic disease; thus, most of the patients did not have data at baseline. Moreover, our patient population was relatively small. Adding this to the low percentage of patients undergoing adjuvant atezolizumab, conclusions about post-operative atezolizumab could not be drawn.

While our data also showed that grade 3 AEs were higher than expected based on similar previous trials, they were manageable and resolved with appropriate interventions. More importantly, there were no AEs that led to death. Furthermore, in this current period of cisplatin and carboplatin supply chain shortages, an alternative regimen that achieves similar results without either of these agents is very welcome.

## 5. Conclusions

Importantly, there is some evidence suggesting that pathCR may not be the best surrogate for DFS/OS in the context of neoadjuvant immunotherapy. Downstaging has been related to favorable survival [33]; also, previous studies have proposed and analyzed the use of other variables such as pathological partial response/major pathologic response, corresponding to <10% residual cancer cells in the resected specimen [34,35,36]. More recently, more complex immune-related pathologic response criteria (irPRC) have been proposed in the context of immune checkpoint inhibitors; irPRC showed better interobserver consistency than the assessment of the percentage of residual viable tumor cells [36]. While a consensus approach has not been established, this remains an important consideration in interpreting trial outcomes following neoadjuvant immunotherapy.

There are currently several larger ongoing trials where the addition of immunotherapy in the localized setting is promising (Table A1). The development of randomized studies to verify survival and safety outcomes is an ongoing endeavor within the cooperative group system that should be encouraged.

Our trial has sought to better understand the use of ICIs in the neoadjuvant setting and showed that a modified FOLFOX + atezolizumab regimen has an acceptable safety profile. This phase I/II trial supports further investigation, in larger prospective trials, of atezolizumab in combination with other immune checkpoint inhibitors for the treatment of localized esophageal and GEJ adenocarcinomas.

## Figures and Tables

**Table 1:** Characteristics of the study population.

Characteristic	N	%
	20	100
Age (years)	Mean (SD)	
	63.5 (9.3)	
	Median (Min-Max)	
	60.9 (39.9–79.1)	
Sex		
Male	17	85
Female	3	15
Race		
Caucasian	19	95
Other	1	5
Hispanic or Latino		
No	16	80
Yes	4	20
ECOG performance status		
0	8	40
1	12	60
Tumor location		
Esophagus	2	10
GEJ Siewert type I	12	60
GEJ Siewert type II	6	30
Baseline T		
T2	1	5
T3	19	95
Baseline N		
N0	8	40
N1	7	35
N2	4	20
N3	1	5
Baseline stage		
IIB	1	5
III	15	75
IVA	4	20
Adenocarcinoma subtype		
Signet ring cells (SRCs)	6	30
Mucinous and SRCs	2	10
n/a	12	60
Tumor grade differentiation		
Moderately (G2)	4	20
G2–G3	6	30
Poorly (G3)	10	50
Microsatellite status		
Stable	10	50
n/a	10	50
PD-L1		
Positive (CPS ≥ 1)	7	35
Negative (CPS < 1)	2	10
n/a	11	55

G2–G3: moderately to poorly differentiated; MSS: microsatellite stable; NX: unknown number of positive lymph nodes; n/a: data not available.

**Table 2:** Efficacy data.

Post-Operative Results	N	%
ypT		
0	2	10
1	3	15
2	2	10
3	11	55
n/a	2	10
ypN		
0	10	50
1	1	5
2	3	15
3	4	20
n/a	2	10
PathCR (ypT0N0M0)		
No	16	80
Yes	2	10
n/a	2	10
Surgical yp stage		
0	2	10
I	4	20
II	4	20
III	4	20
IVA	4	20
n/a	2	10
yp tumor grade differentiation		
0	2	10
Moderately (G2)	3	15
G2–G3	5	25
Poorly (G3)	8	40
n/a	2	10
Affected margins		
Free	15	75
Positive	3	15
n/a	2	10
Lymphovascular invasion		
No	7	35
Yes	11	55
n/a	2	10
Residual cancer		
≥30%	14	70
<1%	4	20
n/a	2	10

G2–G3: moderately to poorly differentiated; MSS: microsatellite stable; NX: unknown number of positive lymph nodes; n/a: data not available. One patient had early progression and one declined surgery. Two of the twenty patients achieved path CR. The observed path CR rate was 10% with a 95% exact confidence interval of (1.24%, 31.7%).

**Table 3:** Number of patients by adverse event grade and relationship to each drug.

Grade	Total Number of Patients	Relationship	Atezolizumab	5FU	Oxaliplatin
5	0		0	0	0
4	2	Definitely	2	0	0
3	8	Definitely	7	5	5
		Unlikely	1	1	2
		Unrelated	3	3	3

**Table 4:** Number of patients who had grade 3 or 4 AEs related to atezolizumab.

Atezolizumab-Related AEs	Grade 3	Grade 4
Hypertensive crisis	0	1
Pancreatitis	1	1
Fatigue	4	0
Nausea	1	0
Dehydration	1	0
Dyspnea	1	0
Myalgia	1	0
Oral pain	1	0
Elevated TSH	1	0

TSH: Thyroid-stimulating hormone.

## Data Availability

All relevant data are provided within the manuscript and Table A1. The datasets generated during and/or analyzed during the current study are available from the corresponding author on reasonable request.

## Appendix A

**Table A1:** Ongoing clinical trials with localized adenocarcinomas treated pre- and post-surgery by ICIs.

NCT# (Abbreviation)	Phase	Location	Histology	Pre- or Post-Surgery ICI	ICI	Radiation	Chemotherapy
NCT02730546	1/2	GEJ/S	Adeno	Pre	Pembrolizumab	Neo	Carboplatin + Paclitaxel
NCT04929392	2	E/GEJ	Adeno/SCC	Pre	Pembrolizumab + Lenvatinib	Neo	Carboplatin + Paclitaxel
NCT05170503	2	GEJ	Adeno	Pre	Sintilimab	No	Tegafur + Oxaliplatin
NCT04592913 (MATTERHORN)	3	GEJ/S	Adeno	Both	Durvalumab	No	FLOT
NCT03221426 (KEYNOTE-585)	3	GEJ/S	Adeno	Both	Pembrolizumab	No	FLOT or Cisplatin + (Capecitabine or 5FU)
NCT04159974 (RICE)	2	E	Adeno	Pre	Durvalumab ± Tremelimumab	Neo	Standard
NCT03604991 (EA2174)	2/3	E/GEJ	Adeno	Pre	Nivolumab + ipilimumab	Neo	Carboplatin + Paclitaxel

E: esophagus; S: stomach; SCC: squamous cell carcinoma.
